# Axillary artery lesions from humeral neck fracture: A study in relation to repair

**DOI:** 10.3892/etm.2012.775

**Published:** 2012-10-30

**Authors:** QUAN ZHANG, SHILONG WANG, CHAOLIANG TANG, WENJUN CHEN, YE ZHANG, LIN CHEN

**Affiliations:** 1 Departments of Orthopaedic Surgery and; 2 Hand Surgery, Huashan Hospital, Fudan University, Shanghai 200040, P.R. China

**Keywords:** axillary artery, humeral neck fracture, reconstruction, diagnosis

## Abstract

Whether axillary artery injuries associated with proximal humeral fractures must be repaired is uncertain. The present study reports three cases treated with various approaches. In case 1, the left humeral surgical neck was broken, the radial pulse disappeared and the arm temperature was significantly reduced. Computerized tomography angiography (CTA) revealed a 3-cm filling deficiency in the axillary artery. The injured artery was reconstructed with a segment of the greater saphenous vein after the fracture was reduced and fixed with a plate. In case 2, the axillary artery was compressed by the broken humeral segment, which caused the right hand to become cool. The fracture was fixed but the axillary artery embolism was not treated. In case 3, the humeral neck was broken with injury to the brachial plexus, although the patient’s hand remained warm. Digital subtraction angiography (DSA) revealed that the axillary artery was injured badly but there was extensive collateral circulation. The proximal humeral fracture was reduced and fixed without artery reconstruction. The three cases all resulted in a good outcome with the bone healed and limb alive. No ischemic necrosis occurred. However, the neurapraxia did not dissappear completely. Axillary artery injury resulting from humeral neck fracture is a rare but disabling traumatic event. Early diagnosis based on signs of acute ischemia of the arm enables early treatment and a favourable outcome. An angiogram is the best way to diagnose the artery injury and evaluate the condition of the collateral circulation. The injured artery in a cold arm should be repaired, while in a warm arm reconstruction is unnecessary due to rich collateral circulation.

## Introduction

Traumatic lesions of the axillary artery are relatively rare, representing 15 to 20% of the arterial injuries of the upper limbs. Of the traumas, 94% are due to penetrating wounds, while the remaining 6% are caused by blunt traumas following shoulder fracture-dislocations (1). The axillary artery is commonly ruptured in high-energy injuries around the shoulder girdle, which are associated with multiple ruptures of nerves whose incidence ranges between 27 and 44% (2). The injured axillary artery may cause a distal ischemia. Consequently, it is hypothesized that vascular repair by interposition grafting should be the treatment of choice (3–8). For those patients with perfectly viable distal limbs due to an extensive anastomotic network of collateral vessels, it remains uncertain whether the injured axillary artery should be reconstructed. The present study reports three cases of proximal humeral fractures associated with axillary artery injury which were treated with or without artery reconstruction.

## Patients and methods

### Case 1

**Figure 1:**
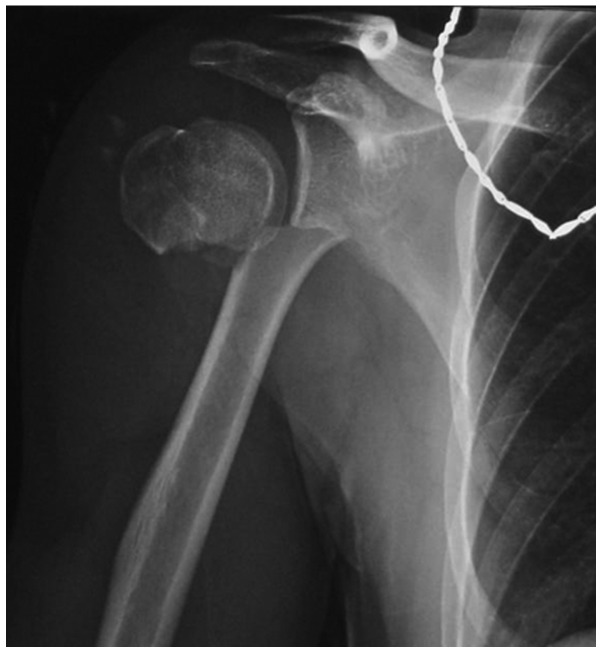
Radiograph of the right shoulder shows a fracture of the humeral surgical neck with medial displacement.

**Figure 2:**
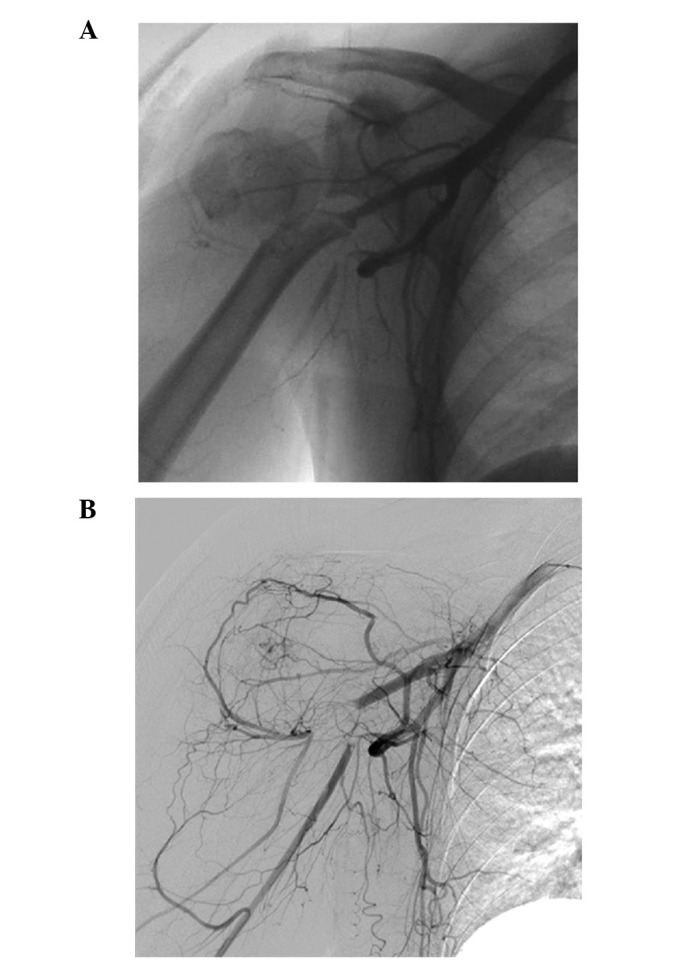
Angiogram of the right-upper extremity. (A) Axillary artery occlusion caused by a distal segment. (B) The injured artery and the extensive collateralization.

**Figure 3:**
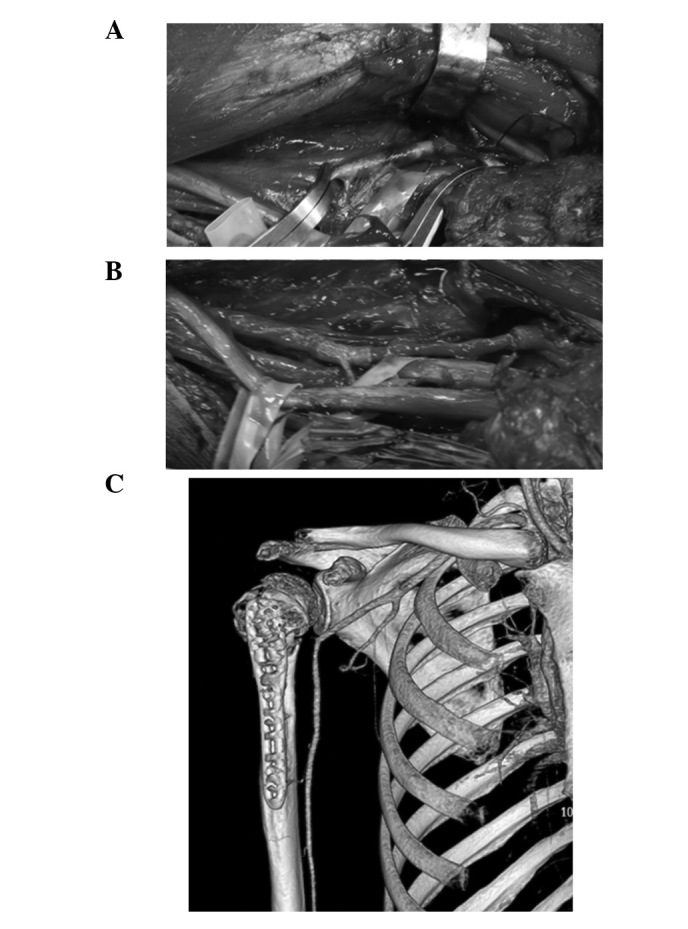
Reconstruction of the injured axillary artery. (A) Intraoperative photograph of the thrombosed axillary artery. (B) Intraoperative photograph showing an interposition graft with a saphenous vein. (C) CTA 3 months after surgery showing repaired artery. CTA, computerized tomography angiography.

A 22-year-old male fell on his outstretched left-upper limb with severe pain in the shoulder area. Two hours after the fall the patient progressively developed coolness, numbness and weakness extending from the fingers to the elbow of the left arm. Physical examination revealed soft-tissue swelling and tenderness around the shoulder. The subclavian pulse was palpable, but the brachial, radial or ulnar pulses were impalpable. The hand and forearm were cool and pale and the capillary refill was slow. The blood pressure of the right arm was 56/46 mmHg, lower than left side which was 116/70 mmHg. Initial radiography of the shoulder revealed a displaced comminuted fracture of the humeral surgical neck (Fig. 1). Computerized tomography angiography (CTA) of the left-upper extremity showed an occluded axillary artery just under the level of glenoid cavity with a 3-cm filling deficiency which was caused by protrusion of the distal segment of the fracture (Fig. 2). The surgical treatment was performed through an extended deltopectoral groove incision. The humeral neck fracture was anatomically reduced and stabilized with a plate and screws. The axillary artery was exposed and observed to be contused and thrombosed between the thoracoacromial artery and circumflex humeral branches (Fig. 3A). The occlusion was caused by an intimal tear with sub-adventitial dissection and secondary thrombosis. The contused segment of the axillary artery was replaced with a greater saphenous vein interposition graft in a reversed fashion (Fig. 3B). The cords of the brachial plexus appeared to be partly injured. Postoperative neurological recovery was incomplete with residual weakness of the wrist and finger extension. The radial pulse was palpable and the blood pressure was almost equal in each arm (right arm was 110/85 mmHg and left arm was 118/82 mmHg). Another CTA confirmed the patency of the arterial repair (Fig. 3C) three months after the operation.

### Case 2

**Figure 4:**
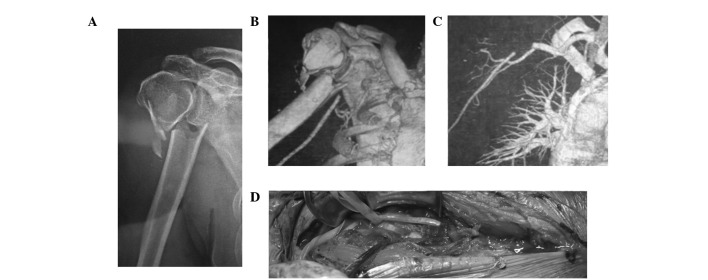
Axillary injury of case 2. (A) Radiograph of the right proximal humerus with a comminuted fracture of the surgical neck. (B) CTA shows the association of the injured artery and the bony segment. (C) CTA shows the thrombosed axillary artery. (D) Intraoperative photograph of the thrombosed axillary artery. CTA, computerized tomography angiography.

A male 45-year-old received a right-humeral surgical fracture during a bicycle accident (Fig. 4A). The patient’s right forearm was cool and two ulnar fingers were numb. The systolic blood pressure of the right arm was 51 mmHg lower than that of left arm. The CTA revealed that the distal humeral segment had trapped the axillary artery, with a 2-cm blood filling deficiency (Fig. 4B). The fracture was reduced and fixed with a lock plate. The axillary artery was explored and an embolism was observed at the contused segment (Fig. 4C). The patient’s permission to repair the artery was not received. As a result, the wound was closed without administering further treatment to the artery. The patient returned home following the surgery. On a follow-up visit three months later, the patient’s right arm was observed to have become significantlay warmer since the patient left the hospital. The same temperature was recorded in both arms. The patient was able to move his hand freely, but the two ulnar fingers remained feeling numb.

### Case 3

**Figure 5:**
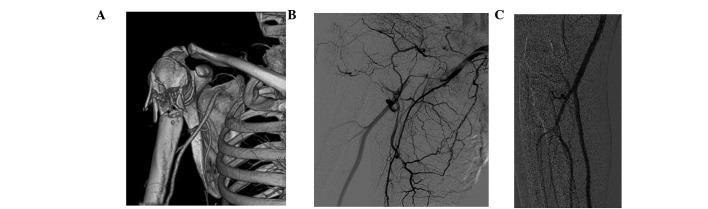
Broken humeral surgery neck and the injured axillary artery of case 3. (A) CTA of the right proximal humerus with a comminuted fracture of the surgical neck and the thinned, discontinued vessel of the first and second segments of the axillary artery. (B) DSA shows the incomplete axillary artery with extensive collateral circulation. (C) DSA shows the brachial, radial and ulnar arteries were engorged well. CTA, computerized tomography angiography; DSA, digital subtraction angiography.

A 47-year-old male construction worker was hit on his right shoulder by a heavy object while working on scaffolding. The humeral neck was broken and the whole arm completely lost motion and feeling (Fig. 5A), although the hand remained warm. The right brachial pulse and radial pulse were palpable, but markedly weaker than than those on the left. The capillary refill was good and the temperature in both arms was the same. However, the systolic blood pressure in the right arm was 60 mmHg, much lower than in the left arm which was 108 mmHg. A Doppler ultrasound scan was performed on the axillary, brachial, radial and ulnar arteries but no vascular injury was observed, although the artery blood-flow speed was slower (19–22 cm/s). Digital subtraction angiography (DSA) revealed that the axillay artery was injured badly at the first and second segments but with extensive collaterals circulation (Fig. 5B) and the brachial, radial and ulnar arteries were engorged (Fig. 5C). This case was discussed carefully with regard to the vessel surgery and neurosurgery and the decision was made not to reconstruct the axillary artery due to three reasons: i) the patient had a warm hand and good capillary refill, meaning that the collateralization was rich; ii) the artery was injured by plucking just below the clavicle and blunted without hematoma. It may have been dangerous to repair it and the iatrogenic trauma would have been large; iii) the patient had total brachial plexus injury from the root, resulting in motion and sensory defects. The function of the arm may not have been able to recover. The proximal humeral fracture was reduced and fixed without artery reconstruction.

## Results

The present study reports three cases of axillary artery injury associated with humeral neck fractures. The patients were all male and injured in right-upper extremity. The mean age was 38 years (22–47 years). In two cases, the axillary artery was injured at the third segment due to the bony segment, while in one case it was injured at the first and second segments which may have been caused by hard plucking. All the fractures were reduced and fixed with plates and healed completely. In one patient (case 1) the axillary artery was repaired with a greater saphenous vein interposition graft in a reversed fashion. Following surgery, the pulse, temperature and blood pressure in the injured arm recovered completely to those of the normal side. In the remaining two cases, the axillary artery lesion was not repaired. The injured arm became warmer in the long-term and no patients developed ischemia. However, the radial pulse was markedly weaker and the blood pressure was notably lower. As for the brachial plexus lesions, all three patients were left with motor or sensory deficiencies.

## Discussion

Vascular injuries of the upper extremity occur in 30 to 50% of vascular extremity traumas (9–11). There is a rare subgroup of patients with an associated vascular injury to the axillary artery and an incidence of 5%. Penetrating trauma is the primary cause of upper extremity vascular injury (90 to 95% of cases). Blunt trauma due to motor vehicle accidents, industrial accidents and falls account for the remaining 5 to 10% (3). The location of the axillary artery, surrounded by the bones and muscles of the shoulder girdle, explains the low incidence of trauma suffered by this arterial segment. Axillary artery injury due to blunt trauma resulting in proximal humeral fractures is even more uncommon. Humeral neck fractures with hyper abduction and traction injuries to the shoulder are a well known cause. The axillary artery is divided into three segments by the pectoralis minor muscle. The first segment of the axillary artery is often injured by clavicular fractures and severe hyper abduction and traction of the shoulder. The second segment is often injured by this type of injury. The injury reported in case 3 belonged to this group of injuries and the first and second segments were damaged. The majority (89%) of traumatic injuries to the axillary artery occur at the level of the third segment of the vessel (12). It is considered to be due to the fact that this segment of the artery is tethered by the anterior and posterior circumflex vessels and it is more susceptible to contusion and laceration from the bony fragments of the humeral neck. The contusion is able to cause an intimal tear with secondary thrombosis. A hematoma dissecting the arterial wall has also been described previously (13) and a similar finding was observed in two of the cases (cases 1 and 2), which were injured by the contusion of the humeral surgical neck and caused a short-segment occlusion in the second and third segments of the axillary artery.

Axillary artery injury due to humeral neck fractures may be identified early with a physical examination. Yagubyan and Panneton (3) reported that pulses were abnormal in 89% involved limbs and 75% had no distal pulse, whereas 14% had decreased pulses. Two of the three patients (cases 1 and 2) had no radial pulse and case 3 had a weak radial pulse. The temperature of the limb was another important physical sign. The majority of the patients with axillary artery injuries reported previously had cold or cool upper extremities (3–10). These patients also exhibited pale fingers and poor capillary refill. Of the limbs of the present cases, case 1 was cold, case 2 was cool and case 3 was as warm as the uninjured arm. The the brachial blood pressure was recorded. All three cases had lower systolic blood pressure than the uninjured arm. The right systolic blood pressure of case 1 was difficult to measure and was 60 mmHg lower. In case 2, it was 51 mmHg lower. Even in case 3 the systolic blood pressure was also was 48 mmHg lower despite the normal temperature. So if a patient with a humeral surgical neck fracture has a cool, pulseless, lower blood pressure arm, this suggests that the axillary artery may be injured and further examination should be performed.

Doppler ultrasound, DSA and venous contrast CTA are useful for to making a definitive diagnosis of axillary artery injuries (3,8,13). Ultrasound requires no wound, is convenient and cheap and is widely used to detect the integrity of the vessels (6). However, it may sometimes provide false-negative results if the patient has rich collateral circulation and it may not detect the right injured segment of the axillary artery due to the fracture. In case 3, the embolized segment of the axillary artery was not identified with ultrasound, but the DSA clearly showed that the axillary artery was injured and a filling deficiency had occurred at the third segment. CTA was used in all three cases. It easily identified the location and the degree to which the artery was damaged. It also clearly showed the association between the artery and the bony segment. In case 1, CTA was used to evaluate the result of the injured artery being replaced by a greater saphenous vein segment. Vessel angiography is usually considered to be necessary in performing a diagnosis of the axillary artery injury (3–6,8).

The broken humeral surgical neck and injured axillary artery may be treated at the same time. The deltopectoral groove approach is advocated, since it exposes the humeral neck and the axillary artery excellently. As in case 1, using a deltopectoral groove incision the fractured fragments of the humeral neck were reduced and fixed with a locked plate and screws. The injured artery was exposed and repaired with a greater saphenous vein interposition graft. In cases of axillary arteries with blunt injuries, the orthopedic procedure is often required before vascular repair is undertaken since the reduction and stabilization of the humeral neck fracture may allow the vessel repair to be performed conveniently and prevent the repaired artery from being injured again.

Whether the injured axillary artery should be reconstructed is not clear. It is usually (3–8,11,13,14) considered to be necessary to repair the artery as soon as possible and even in those with adequate distal perfusion, fracture stabilization and vascular repair is likely to improve the long-term outcome and posttraumatic cold intolerance (15). Conservative management is clearly advised against in an ischemic limb (13). When the artery is broken or blocked, the distal segment of the limb has a lower systolic blood pressure, meaning that the tissue perfusion pressure is lower and ischemia is likely to occur. In the present cases of axillary artery injuries, all had lower blood pressures. Case 1 underwent a formal arterial reconstruction and the upper extremity recovered normal temperature, strong radial pulse, normal blood pressure and good capillary refill. This is beneficial for injured limb recovery. When reviewing the literature, >70% of the patients underwent reconstruction, including end-to-end anastomosis and interposition or bypass grafts with saphenous veins or vascular prostheses. A simple primary repair involving intimal excision and tacking down with or without a thrombectomy was performed in a number of patients. Shalhub *et al* describe an endovascular technique utilizing combined brachial and femoral access to create a through-and-through brachial-femoral wire and repair the arterial injury with a covered stent (16). Only ∼10% of the patients were treated conservatively, including an elderly patient with an old fracture. The injured limb may be alive due to the presence of extensive collateral circulation (3).

The axillary artery has 5 major branches which form multiple anastomoses and develop a web-like network, supplying the thoracic and scapular/humeral areas and providing excellent collateral circulation around the shoulder girdle. A short-segment occlusion of the axillary artery between the origins of these arterial branches that prevents the flow through them may be effectively bypassed by these collaterals. A discussion with the vascular surgeons suggested that the vascular injury may be treated conservatively by relying on collateral circulation, as the limb was warm and the brachial plexus injury was not due to a pressure effect from a large hematoma (8). In cases 2 and 3, although the axillary arteries were occluded or ruptured and the patients did not undergo reconstruction, the extremity did not experience ischemia in the long-term due to the rich collateralization. The artery in case 3 was not reconstructed based on three factors: i) the patient had a warm arm and good capillary refill, meaning that the collateralization was rich; ii) the artery was injured by plucking just below the clavicle and blunted without hematoma. It may have been dangerous to repair it and the iatrogenic trauma would have been large; iii) the patient had total brachial plexus injured from the root, resulting in motion and sensory defects. The function of the arm may not have been able to recover. This case was discussed carefully with regard to the vessel surgery and the decision was made to reduce and fix the fracture only. Although two of the present cases and a number reported previously (3) had non-ischemic limbs without artery reconstruction, it is necessary to evaluate the long-term outcome.

The intimate association of the brachial plexus and the axillary artery is so close that it is a notable feature of axillary artery trauma associated with brachial plexus injury (3,7). The brachial plexus and axillary artery lie in a common fascial sheath. Damage to the artery, which may cause only minimal swelling, is capable of leading to early compression of the brachial plexus and its components. The main cause of injury is the direct damage resulting from the broken humeral bone segment. Axillary sheath hematomas may be another reason for delayed ischemia. Of these patients, 46% exhibited a neurological deficit (17). Johnson *et al*(17) reported a 43.5% incidence of brachial plexus injury occurring in patients with subclavian and axillary artery vascular trauma. Other studies have also reported a similarly high incidence of nerve injury in upper extremity vascular trauma, between 35 and 70% (3,18–21). In the present cases, all patients had different brachial complex injuries and were left with neurological motion and sensory defects.

Axillary artery injury resulting from humeral neck fractures is a rare but disabling traumatic event (3). Special attention should be payed to fractures with abduction and severe medial displacement. The patients’ pulse, temperature and blood pressure are highly sensitive physical signs of axillary artery injuries. Angiograms, including DSA and CTA, are the best method for diagnosing arterial injuries and evaluating the condition of the collateral circulation. The majority of these patients should undergo artery reconstruction while the humeral neck fracture is reduced and fixed, with the exception of certain special cases. Recognition of concomitant brachial plexus injury is also important for decreasing the neurological morbidity. The orthopedic surgery, vessel surgery and neurosurgery should work together to treat these patients (21).
